# Anaesthesia for Caesarean Section in a Parturient with Klippel-Feil Syndrome: A Case Report

**DOI:** 10.4274/TJAR.2026.252304

**Published:** 2026-06-26

**Authors:** Ivana Bureš Valentić, Krešimir Reiner

**Affiliations:** 1 University Hospital Centre Zagreb, Department of Anaesthesiology and Reanimatology, Intensive Care and Pain Management, Zagreb, Croatia; 2 University of Zagreb Faculty of Medicine, Department of Anaesthesiology and Reanimatology, Intensive Care and Pain Management, Zagreb, Croatia

**Keywords:** Klippel-Feil syndrome, caesarean section, spinal anaesthesia, difficult airway, obstetric anaesthesia

## Abstract

Klippel-Feil syndrome (KFS) is a rare congenital condition defined by the fusion of one or more cervical vertebrae, often associated with a range of skeletal and extra-skeletal abnormalities. The presence of cervical vertebral fusion and spinal deformities can make both airway management and neuraxial anaesthesia technically challenging in this population. This report describes a primigravida with KFS who underwent an elective caesarean section under spinal anaesthesia. The preparation included consideration of a potentially difficult airway, even though general anaesthesia had not initially been planned. We reviewed relevant literature on anaesthetic management in similar cases. There is no consensus on the optimal anaesthetic technique for the management of parturients with KFS undergoing caesarean section. Each case should be evaluated individually. It is essential to prepare for potential conversion to general anaesthesia and always prioritize patient safety.

**Main Points**

• Patients with Klippel-Feil syndrome (KFS) have a difficult airway and spinal anatomy, which make both airway management and neuraxial anaesthesia challenging.

• Spinal anaesthesia can be used successfully in these patients, but preparation for a possible conversion to general anaesthesia is essential.

• Individualized anaesthetic plans are required, as no single technique is recommended for all KFS patients.

## Introduction

Klippel-Feil syndrome (KFS), first described in 1912, is a rare congenital disorder characterized by the fusion of one or more cervical vertebrae, often accompanied by skeletal and extra-skeletal anomalies.^1^ While some cases follow autosomal dominant or recessive inheritance, most are sporadic. Feil originally classified KFS into three variants based on the extent of spinal fusion.^2^ Frequently associated anomalies include scoliosis, renal abnormalities, Sprengel deformity, deafness, congenital heart disease, and synkinesis.^2, 3, 4, 5, 6, 7, 8, 9, 10, 11, 12^ Patients with KFS may present for various surgical procedures, including caesarean section. Cervical spine fusion and restricted neck mobility increase the risk of a difficult airway. Airway management in pregnancy is particularly challenging, emphasizing the need for detailed assessment.^12, 13, 14^ Neuraxial anaesthesia may also be difficult due to abnormal spinal anatomy, increasing the risk of a failed or unpredictable block.^15^ We report the successful anaesthetic management of a primigravida with KFS undergoing an elective caesarean delivery.

## Case Report

**Figure 1:**
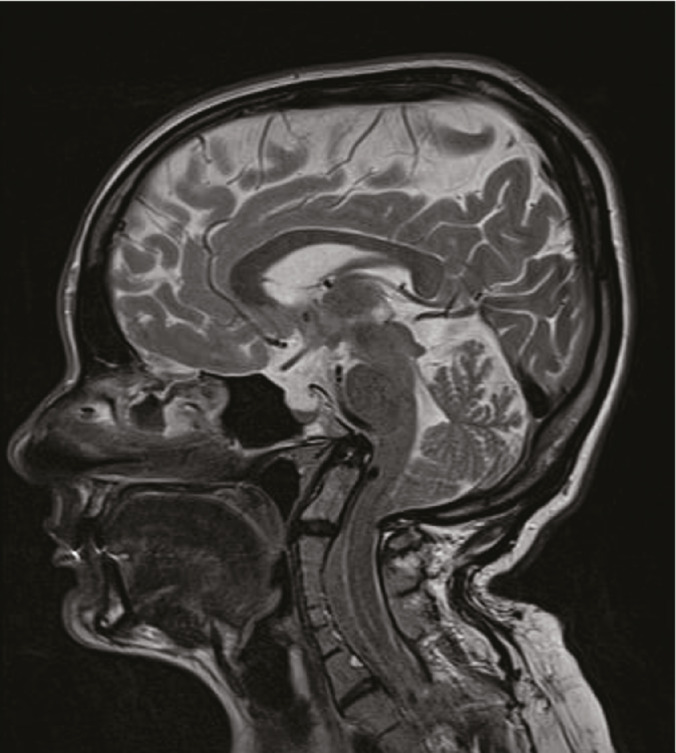
Pre-pregnancy magnetic resonance imaging showing fusion of vertebrae C3 to Th1.

A 32-year-old primipara with KFS was admitted at 40 weeks’ gestation for surveillance and preparation for an elective caesarean section. Preoperative evaluation included medical history review, airway assessment, and spine imaging. Her KFS features included cervical fusion from C3 to T1, kyphoscoliosis, bilateral Sprengel deformity, and right-sided deafness. Overall, these findings are consistent with type 1 according to Feil classification.^2^ She also had polycystic ovary syndrome. Family history was unremarkable for genetic or anaesthetic complications. During childhood, she underwent surgical correction of both scapulae. More recent procedures included a tonsillectomy and a right mastoidectomy, which were performed under general anaesthesia with videolaryngoscopy and a reinforced 6.0 endotracheal tube. Although intubation was not documented as difficult, bag-mask ventilation was difficult during one induction. She took no chronic medications, reported no allergies, and remained physically active prior to pregnancy. Her pregnancy was uneventful, except for mild exertional dyspnea and a newly observed left-sided cervical fat nodule. On examination, her height and weight were 154 cm and 81 kg, respectively (body mass index 34.2 kg m^2-1^). She had a short neck with limited range of motion. Airway assessment revealed a Mallampati II classification and an adequate mouth opening. Palpation of the lumbar spine was unremarkable despite thoracic kyphoscoliosis. Postoperative scarring was present at the level of the thoracic spine. Laboratory studies demonstrated mild anemia (hemoglobin 102 g L^-1^) and normal coagulation. Pre-pregnancy magnetic resonance imaging showed platybasia, basilar invagination, and significant cervical deformity (Figure 1), with a normal spinal canal width below the craniocervical junction. A neurosurgical review supported the feasibility of neuraxial anaesthesia.

The risks and benefits of regional anaesthesia were discussed, and informed consent was obtained. In the operating theatre, standard monitoring was applied, and a difficult airway cart containing equipment for emergency ventilation and intubation, including supraglottic devices, a videolaryngoscope, and a flexible bronchoscope, as well as a surgical cricothyrotomy kit, was prepared. After placement of intravenous access, aspiration prophylaxis with metoclopramide 10 mg was administered. The patient was positioned in a sitting position. Following two unsuccessful attempts, the subarachnoid space was accessed at L3-L4 using a 27-gauge Whitacre needle. Spinal anaesthesia was achieved with 10 mg of 0.5% hyperbaric bupivacaine and 20 µg of fentanyl. After block placement, she was positioned supine with left uterine displacement. High-flow nasal oxygenation (HFNO) was provided to optimize oxygen reserve in case conversion to general anaesthesia became necessary.

A sensory level to pinprick at T6 was confirmed before incision. Since the patient tolerated the supine position and maintained adequate respiratory function, the high-flow nasal cannula was later removed. Caesarean delivery proceeded uneventfully, resulting in a healthy male infant weighing 3500 g, with Apgar scores of 9 and 10 at 1 and 5 minutes, respectively. Antibiotic prophylaxis (cefazolin, 2 g) and oxytocin infusion (20 IU over four hours) were administered. Hemodynamics remained stable throughout the procedure. The patient was transferred to the post-anaesthesia care unit for continued monitoring and analgesia for 24 hours. Overall, the postoperative course was uneventful. Visual analogue pain scale was monitored for every 2 hours and analgesia was titrated to keep visual analogue pain scale in the mild range (≤3-4). Over the first 12 postoperative hours, our patient received intravenous tramadol by continuous infusion (300 mg in total), and intravenous paracetamol (1 gram) combined with ibuprofen (300 mg) every six hours. After the patient resumed normal fluid intake and was able to sit in bed approximately 12 hours after caesarean, analgesia was converted from parenteral to peroral.

## Discussion

Neuraxial anaesthesia is considered the gold standard for caesarean delivery, with use exceeding 80% in some countries.^16, 17^ For patients with known or suspected difficult airways, regional techniques are generally preferred, particularly during pregnancy. However, anatomical anomalies may complicate neuraxial block placement. Spinal anaesthesia, even when successful, does not eliminate the possibility of airway intervention, including emergent conversion to general anaesthesia.^18^ Accordingly, guidelines emphasize preparation, equipment readiness, and team communication.^14^ HFNO was used proactively to extend safe apnea time should intubation become necessary, although evidence on its use in the pregnant population remains limited.^19, 20^

Previous reports of anaesthetic management in KFS parturients describe varied strategies depending on clinical features.^3, 4, 5, 6, 7, 8, 9, 10, 11, 12^ Kavanagh et al.^3^ outlined the advantages and limitations of several approaches, each carrying distinct risks. Structural spinal pathology is relatively common in pregnancy and may challenge neuraxial administration due to difficulty identifying landmarks, altered ligamentous structures, and unpredictable drug spread.^15, 21, 22^

Our anaesthetic choice was influenced by concerns about potential cervical spine instability and abnormal atlanto-occipital anatomy, which may increase the risk of neurological injury during airway manipulation. Spinal anaesthesia allowed the patient to maintain control of her own neck positioning and to communicate discomfort. Other available techniques, such as epidural and combined spinal-epidural anaesthesia, were also considered as potential options in our patient. However, epidural catheter placement was deferred primarily because of concern that a large volume administered epidurally may increase intracranial pressure, which is particularly notable in patients with craniovertebral junction abnormalities. Furthermore, epidural catheter placement can be technically more demanding when anatomy is distorted and carries a higher risk of a failed block. Therefore, we decided to administer spinal anaesthesia, which has been shown to be a reasonable and straightforward choice because of its rapid onset, reliability, and ease of performance without significant technical challenges.^3, 4, 5, 6, 7, 8, 9, 10^ A mixture of hyperbaric bupivacaine and fentanyl was utilized, with the dose of bupivacaine adjusted to patient’s height and weight, as this dosing approach has been shown to provide adequate anaesthesia for elective caesarean section and is associated with a lower incidence of maternal hypotension.^23^

Even though we had ultrasound available in the operating theatre as a backup method, its application was not necessary in our case due to adequate anatomical landmarks and successful identification of the subarachnoid space on the second attempt. However, it is to highlight the advantages of ultrasonography as a guidance tool in patients with challenging anatomy or obesity.^24^

## Conclusion

No consensus exists regarding the optimal anaesthetic management for parturients with KFS undergoing caesarean delivery. Each case requires individualized planning based on anatomy, clinical expertise, and resource availability. Preparation for potential conversion to general anaesthesia remains essential, with patient safety prioritized.

## Ethics

**Informed Consent:** Informed consent was obtained.
